# Modulating *Salmonella* Typhimurium's Response to a Changing Environment through Bacterial Enhancer-Binding Proteins and the RpoN Regulon

**DOI:** 10.3389/fmolb.2016.00041

**Published:** 2016-08-17

**Authors:** Christine E. Hartman, David J. Samuels, Anna C. Karls

**Affiliations:** Department of Microbiology, University of GeorgiaAthens, GA, USA

**Keywords:** *Salmonella* RpoN regulon, sigma 54, bacterial enhancer-binding protein, bEBP, transcription activation, stress adaptation

## Abstract

Transcription sigma factors direct the selective binding of RNA polymerase holoenzyme (Eσ) to specific promoters. Two families of sigma factors determine promoter specificity, the σ^70^ (RpoD) family and the σ^54^ (RpoN) family. In transcription controlled by σ^54^, the Eσ^54^-promoter closed complex requires ATP hydrolysis by an associated bacterial enhancer-binding protein (bEBP) for the transition to open complex and transcription initiation. Given the wide host range of *Salmonella enterica* serovar Typhimurium, it is an excellent model system for investigating the roles of RpoN and its bEBPs in modulating the lifestyle of bacteria. The genome of *S.* Typhimurium encodes 13 known or predicted bEBPs, each responding to a unique intracellular or extracellular signal. While the regulons of most alternative sigma factors respond to a specific environmental or developmental signal, the RpoN regulon is very diverse, controlling genes for response to nitrogen limitation, nitric oxide stress, availability of alternative carbon sources, phage shock/envelope stress, toxic levels of zinc, nucleic acid damage, and other stressors. This review explores how bEBPs respond to environmental changes encountered by *S*. Typhimurium during transmission/infection and influence adaptation through control of transcription of different components of the *S*. Typhimurium RpoN regulon.

## Introduction

*Salmonella enterica subsp. enterica* serovar Typhimurium is the most common serotype of *Salmonella enterica* subspecies, which causes tens of millions of cases of salmonellosis and more than 100,000 deaths worldwide each year (Majowicz et al., 2010). *S*. Typhimurium has been extensively studied to reveal the virulence factors and strategies that lead to morbidity and mortality, defining novel mechanisms of bacterial transmission and pathogenesis (reviewed in Fàbrega and Vila, 2013). The response of *S*. Typhimurium to the stresses it encounters in its infectious pathway—from the external environment to the host's intestines—is controlled largely by overlapping transcriptional regulatory systems (reviewed in Runkel et al., 2013).

Transcription in bacteria is carried out by the RNA polymerase core enzyme (RNAP; α_2_ββ′ω). However, the core enzyme alone cannot recognize specific promoter sequences; the variable sigma (σ) subunit confers DNA-binding specificity to ensure that transcription starts at the appropriate promoter sequence (reviewed in Feklístov et al., 2014). RNAP and σ together make up the holoenzyme (Eσ). There are two families of sigma factors: the σ^70^ (RpoD) family and the σ^54^ (RpoN) family. The σ^70^ family includes the housekeeping sigma factor (σ^70/D^) and all of the alternative sigma factors, except σ^54^. These σ^70^-type sigma factors, which in *Salmonella* include σ^70/D^, σ^24/E^, σ^32/H^, σ^38/S^, and σ^28^, exhibit similar structure and recognize promoter sequences with −35 (TTGACA) and −10 (TATAAT) promoter elements that are conserved to varying extents. When Eσ^70^ binds to promoter sequences, it initially forms a closed complex, where no DNA melting has occurred. Free energy from specific interactions of Eσ^70^ with promoter DNA activate conformational changes in both Eσ^70^ and DNA to form a stable open complex in which duplex DNA is opened at the +1 transcription start site and the template strand moves into the active site of RNAP (reviewed in Saecker et al., 2011).

**Figure 1:**
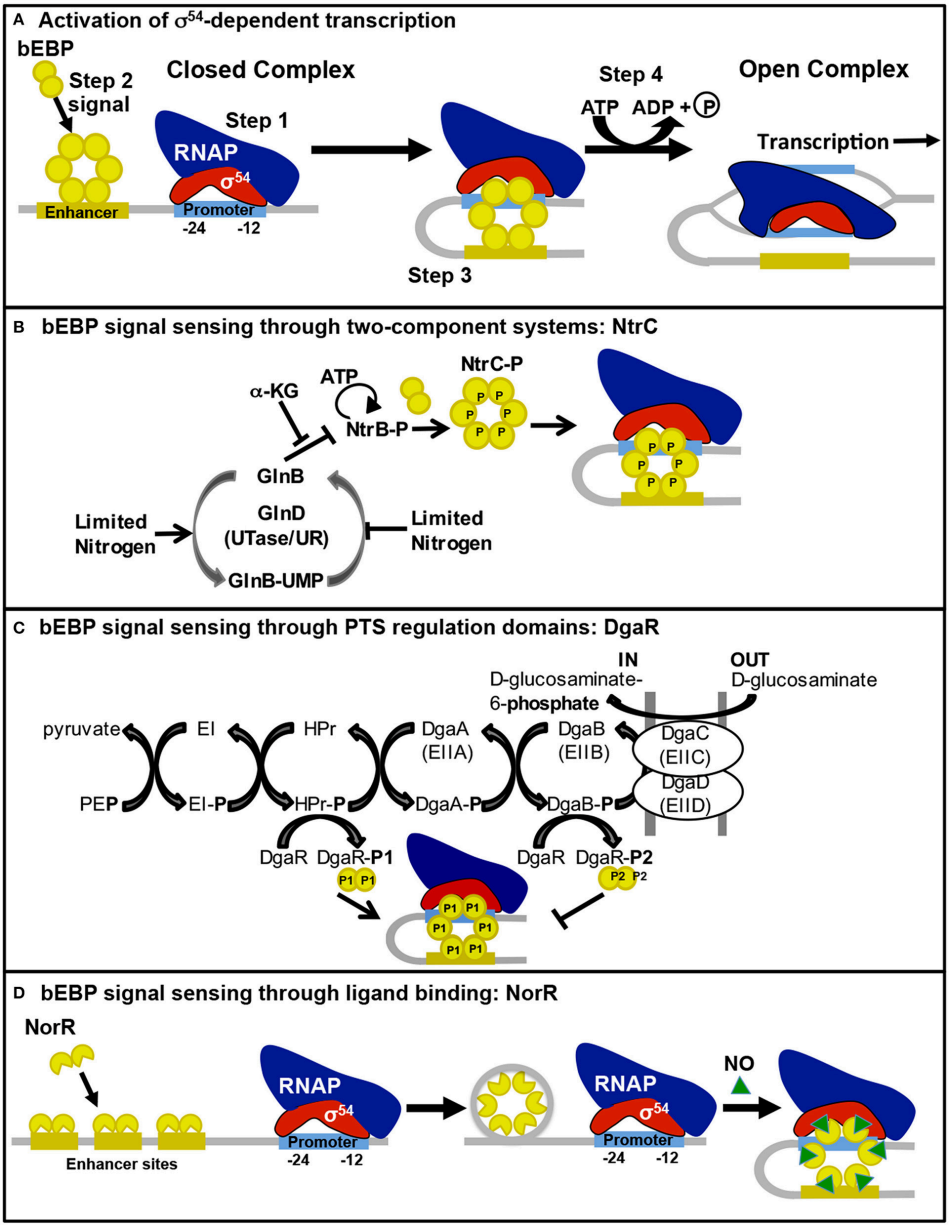
**Bacterial enhancer-binding protein sensing of environmental signals and activation of σ^54^-dependent transcription**. The process for bEBP activation of σ^54^-dependent transcription is illustrated in **(A)**. Step 1, Eσ^54^ binds to the promoter in a stable closed complex. Step 2, the bEBP receives a signal from the internal or external environment, becomes active, and binds to an enhancer sequence. Step 3, DNA looping brings the bEBP in contact with Eσ^54^. Step 4, the bEBP hydrolyzes ATP to promote open complex formation. The mechanism for bEBP sensing of environmental signals through **(B)** two-component systems, **(C)** PTS regulatory domains, and **(D)** ligand binding are illustrated here and described in the text.

σ^54^ is structurally distinct from the σ^70^-type sigma factors (Yang et al., 2015), thus Eσ^54^ recognizes very different promoter elements located at −24 (GC) and −12 (GG) upstream of the transcription start site (Morett and Buck, 1989). When Eσ^54^ binds to a promoter, it forms a stable closed complex due to direct interaction of Eσ^54^ with two bases within a DNA distortion immediately downstream of the −12 element (Morris et al., 1994). Open complex formation by Eσ^54^ requires an activator protein (bacterial enhancer-binding protein; bEBP; Yang et al., 2015). bEBPs are typically found as dimers in the cell but, upon receiving the appropriate cellular signal, they oligomerize into complexes that are competent to bind ATP and interact with enhancer sequences usually located 80–150 bp upstream of the promoter (Figure 1A). A DNA-looping event, often facilitated by integration host factor, brings the bEBP oligomer in contact with Eσ^54^ at the promoter (Wedel et al., 1990); bEBP then hydrolyzes ATP, which causes conformational changes in bEBP that trigger remodeling of Eσ^54^ and stimulate open complex formation (Chen et al., 2010). Bacteria often have multiple bEBPs that are responsive to different environmental signals and activate transcription of different sets of genes (Francke et al., 2011).

The global RpoN regulon of *S*. Typhimurium, including σ^54^-dependent transcripts and Eσ^54^ chromosomal DNA-binding sites, was characterized in the presence of a promiscuous, constitutively-active bEBP using microarray and ChIP-chip analyses (Samuels et al., 2013). Promoters of this extensive and diverse RpoN regulon in *S*. Typhimurium respond to 1 of 13 known or predicted bEBPs (Table 1; Studholme, 2002). The target promoters and activating environmental stimuli for most of these bEBPs have been demonstrated experimentally or inferred from studies with orthologs in *E. coli* (Table 1). RpoN regulons of *S.* Typhimurium (Samuels et al., 2013) and *E. coli* (Zhao et al., 2010; Bonocora et al., 2015) share many genes/operons (see Table 1); significant differences include the absence in *Salmonella* of *nac*, the LysR-type regulator of multiple operons involved in nitrogen assimilation (Zimmer et al., 2000), and the absence in *E. coli* of the *gfr* operon and *rsr-yrlBA* of the *Salmonella* RNA repair operon (see below). Cellular processes regulated by σ^54^-dependent bEBPs in *S.* Typhimurium include nitrogen metabolism in response to limiting nitrogen conditions [NtrC (GlnG, NRI), Keener and Kustu, 1988; Zimmer et al., 2000], transport and catabolism of D-glucosaminate (DgaR, Miller et al., 2013) and glucoselysine/fructoselysine (GfrR, Miller et al., 2015), regulation of cytoplasmic pH homeostasis during fermentative growth by the formate-hydrogen lyase system (FhlA, Hopper and Böck, 1995; Lamichhane-Khadka et al., 2015), response to assaults to the cell envelope (PspF, Karlinsey et al., 2010; Flores-Kim and Darwin, 2015 and zinc-dependent ZraR, Appia-Ayme et al., 2012), reduction of nitric oxide under anaerobic conditions (NorR, Hutchings et al., 2002; Mills et al., 2005), propionate catabolism (PrpR, Palacios and Escalante-Semerena, 2000), regulation of amino-sugar synthesis by sRNAs (GlrR; Gopel et al., 2011), and RNA repair/processing (RtcR, Samuels, 2014; Engl et al., 2016). A comprehensive study of the genes that are required for infection of animal hosts by *S*. Typhimurium identified RpoN as important in colonization of chicks, pigs, cattle and mice; transposon mutants in bEBP genes *ntrC* (*glnG*) and *prpR* were attenuated in at least two animal hosts and RpoN-regulated genes *argT, glnA, glnL*, and *gfrACDEF* (SL1344_4466, 4468–4471) were attenuated in at least two animal hosts (Chaudhuri et al., 2013).

**Table 1:** **σ^54^-dependent genes and associated bEBPs in *S.* Typhimurium^a^**.

**Locus tag^b^**	**Gene symbol**	**Function**	**bEBP^c^**	**bEBP enhancer sequence^d^**	**Activating signal/condition^e^**
STM0368-71	*prpBCDE*	Propionate catabolism	PrpR	CGTTTCATGAAACG	2-methylcitrate
STM0462	*glnK amtB*	Regulator of N metabolism; NH_3_ transporter	NtrC	TGCACC(A/T)_4_TGGTGCA	Low intracellular glutamine
STM0665-62^f^	*gltIJKL*	Glutamate/aspartate transporter	NtrC	TGCACC(A/T)_4_TGGTGCA	Low intracellular glutamine
STM0830-28	*glnHPQ*	Glutamine high-affinity transporter	NtrC	TGCACC(A/T)_4_TGGTGCA	Low intracellular glutamine
STM1285-84^f^	*yeaGH*	Serine protein kinase	NtrC	TGCACC(A/T)_4_TGGTGCA	Low intracellular glutamine
STM1303-07	*astCABDE*	Arginine/ornithine/glutamine matabolism	NtrC	TGCACC(A/T)_4_TGGTGCA	Low intracellular glutamine
STM2355	*argT*	Lysine/arginine/ornithine transport protein	NtrC	TGCACC(A/T)_4_TGGTGCA	Low intracellular glutamine
STM4007-05	*glnALG*	Glutamine synthetase	NtrC	TGCACC(A/T)_4_TGGTGCA	Low intracellular glutamine
STM0577-72	-----	Putative PTS	STM0571	NK	NK
STM0649-51	-----	Putative hydrolase, 2-keto-3-deoxygluconate permease	STM0652	NK	NK
STM1690-86	*pspABCDE*	Phage shock proteins	PspF	TAGTGTAATTCGCTAACT	Cell envelope stress
STM4244^f^	*pspG*	Phage shock protein	PspF	TAGTGTAATTCGCTAACT	Cell envelope stress
STM2360-56	-----*ubiX*	Amino acid transport	STM2361	NK	NK
STM2840-41	*norV ygbD*	Nitric oxide reductase	NorR	GT(N)_7_AC	Nitric oxide
STM2843-42	*hydN hypF*	Putative hydrogenase maturation proteins	FhlA	CATTTCGTACGAAATG	Formate
STM2853-44	*hycABCDEFGHI-*	Hydrogenase 3	FhlA	CATTTCGTACGAAATG	Formate
STM2854-58	*hypABCDE*	Hydrogenase maturation proteins	FhlA	CATTTCGTACGAAATG	Formate
STM3521-18	*rsr yrlBA rtcBA*	Nucleic acid repair/processing	RtcR	NK	Nucleic acid damage
STM3568^f^	*rpoH*	Heat shock sigma factor (σ^32^)	NK	NK	NK
STM3772-66	*dgaABCDEF*	D-glucosaminate utilization	DgaR	NK	D-glucosaminate
STM4172	*zraP*	Zinc-dependent chaperone	ZraR	NK	[Zinc] and cell envelope stress
STM4173-74	*zraSR*	Zinc-responsive two component system	ZraR	NK	[Zinc] and cell envelope stress
STM4285	*fdhF*	Formate dehydrogenase	FhlA	CATTTCGTACGAAATG	Formate
STM4535-40	*gfrABCDEF*	Glucoselysine and fructoselysine utilization	GfrR	NK	Glucoselysine, fructoselysine
STM_R0152^g^	*glmY*	GlmY sRNA	GlrR	TGTC(N)_10_GACA	NK
STM_R0167^g^	*glmZ*	GlmZ sRNA	GlrR	TGTC(N)_10_GACA	NK

a *Eσ^54^ binding to promoters for all indicated operons was confirmed in S. Typhimurium by ChIP-chip (Samuels et al., 2013); σ^54^-dependent expression of all genes in S. Typhimurium was confirmed by microarray (Samuels et al., 2013), with the few exceptions that are footnoted*.

b *Locus tags for σ^54^-dependent genes in S. Typhimurium LT2 are underlined if found in E. coli (solid line if found in most sequenced strains; dashed line if found in few E. coli strains; dotted line if only part of the operon is found in E. coli)*.

c *Known or predicted bacterial enhancer-binding protein (bEBP) that activates the σ^54^-dependent gene or operon (see text for references). NK, not known*.

d *Consensus enhancer sequence given for each bEBP is based on enhancers associated with one or more of the target promoters in one or more bacterial genus; references: PrpR (Palacios and Escalante-Semerena, 2004), NtrC (Ferro-Luzzi Ames and Nikaido, 1985), PspF (Lloyd et al., 2004), NorR (Tucker et al., 2004), FhlA (Leonhartsberger et al., 2000), and GlrR (Gopel et al., 2011). NK, not known*.

e *Specific signal or condition that results in activation of the bEBP (see text for references). NK, not known*.

f *Evidence for expression from the σ^54^-dependent promoter in Salmonella has not been published*.

g *σ^54^-dependent expression in Salmonella was shown in Gopel et al. (2011)*.

## Bacterial enhancer-binding proteins of *S*. typhimurium sense and respond to signals for adaptation in a changing environment

bEBPs typically consist of three domains: an N-terminal regulatory domain, a central AAA+ ATPase/transcriptional activation domain, and a C-terminal DNA-binding domain. The N-terminal regulatory domain responds to cellular signals and negatively or positively controls AAA+ domain oligomerization, ATPase activity, and/or interaction with σ^54^. The central AAA+ ATPase domain is responsible for bEBP oligomerization; association of two AAA+ domains within the bEBP oligomer forms the ATP hydrolysis site. This domain also includes the highly conserved GAFTGA motif that mediates the interaction with σ^54^. The C-terminal DNA-binding domain contains a helix-turn-helix DNA-binding motif, which determines bEBP specificity for an enhancer. For some bEBPs binding to the enhancer facilitates or stabilizes oligomerization. Consensus enhancer sequences for bEBPs found in *S*. Typhimurium are given in Table 1. Further details on bEBP structure and function are reviewed in (Bush and Dixon, 2012).

The regulatory domains of *S.* Typhimurium bEBPs can function as response-regulator domains of two-component systems (TCS), phosphotransferase regulation domains (PRDs) or ligand-binding domains. One bEBP, PspF, lacks a regulatory domain, but a separate protein, PspA, controls PspF activity. The PspF-PspA system, which is required for *S*. Typimurium virulence in a mouse model (Karlinsey et al., 2010), is not further discussed in this review, but two recent studies provide insight into this anti-activator mechanism for regulating bEBP activity (Flores-Kim and Darwin, 2015; Osadnik et al., 2015). Representative examples for the different mechanisms by which the regulatory domains of bEBPs from *S.* Typhimurium respond to extracellular or intracellular signals are considered here.

### Signal sensing through two-component systems

*S.* Typhimurium has three bEBPs (NtrC, ZraR, and GlrR) that are response regulators of TCSs, in which a sensor kinase protein recognizes the cellular signal, autophosphorylates, and transfers the phosphate to a conserved aspartate residue of the response regulator. Phosphorylation of the regulatory domain stimulates the bEBP to interact with enhancer sequence(s) and the Eσ^54^ closed complex, activating open complex formation (Figure 1B).

#### NtrC (GlnG)

The NtrB-NtrC TCS is activated in response to limited nitrogen conditions. NtrB is the sensor kinase of the TCS. Nitrogen limitation is perceived by the cell as low intracellular levels of glutamine (Ikeda et al., 1996), which stimulates the uridylyltransferase GlnD to uridylylate the P_II_ protein GlnB (Jiang et al., 1998). Unmodified GlnB inhibits NtrB kinase activity but GlnB-UMP cannot interact with NtrB, thus allowing autophosphorylation of NtrB and transfer of the phosphate to NtrC (Reitzer, 2003). GlnB also responds to α-ketoglutarate. During nitrogen limitation, the level of α-ketoglutarate is high and inhibits GlnB interaction with NtrB, thereby increasing NtrC phosphorylation (Schumacher et al., 2013). Phosphorylation of NtrC dimers results in oligomerization and enhancer binding (Weiss et al., 1991). NtrC-dependent transcription of target genes (Table 1) allows the cell to assimilate low levels of ammonia and utilize alternative nitrogen sources in nutrient-limited environments; NtrC-regulated *glnA* (glutamine synthetase) and *glnHQ* (glutamine transport) together contribute to *S.* Typhimurium virulence in a mouse model and increased survival in macrophages (Klose and Mekalanos, 1997).

#### ZraR (HydG)

In *S*. Typhimurium, ZraR is a response regulator, activated by its sensor kinase ZraS in a zinc-dependent response to envelope stress (Leonhartsberger et al., 2001; Appia-Ayme et al., 2012). ZraR controls expression from divergent σ^54^-dependent promoters for *zraSR* and *zraP.* ZraP encodes a zinc-binding periplasmic protein that acts as a zinc-dependent chaperone in both *S.* Typhimurium and *E. coli*; ZraP responds to misfolding of periplasmic and outer membrane proteins due to envelope stress, such as disruption of the outer membrane by antimicrobial cationic peptides that may be encountered in the environment and/or the host (Appia-Ayme et al., 2012; Petit-Härtlein et al., 2015).

### Signal sensing through phosphotransferase regulation domains

The bEBPs DgaR, GfrR, and STM0571 of *S.* Typhimurium are members of the family of LevR-like regulators, which previously have only been described in Gram-positive bacteria controlling transcription of the genes for permease components of phosphotransferase systems (PTSs) and enzymes required for utilization of the imported sugar/amino sugar (reviewed in Deutscher et al., 2014). PTSs import and phosphorylate sugars through the Enzyme II complex (EII) membrane-bound components that are linked to a cascade of phosphoryl transfer, beginning with phosphoenolpyruvate as the donor and continuing through Enzyme I (EI), HPr, and finally the EII complex (Figure 1C). These PTS enzymes control the activity of the LevR-like bEBPs through phosphorylation of the regulatory domain. In contrast to most bEBPs, the regulatory domains of LevR-like bEBPs are found at the C-terminus. These regulatory domains contain two PTS regulation domains (PRDs) with competing activities. HPr-mediated phosphorylation of a conserved histidine residue adjacent to PRD1 leads to activation while EII-mediated phosphorylation of a conserved histidine residue within PRD2 is inhibitory (Martin-Verstraete et al., 1998).

#### DgaR

The LevR-like bEBP DgaR is phosphorylated by PTS HPr~P (DgaR-P1), resulting in expression of *dgaABCDEF*, which encodes the permease and catabolic enzymes for D-glucosaminate (Miller et al., 2013). When D-glucosaminate is present, EII preferentially phosphorylates the sugar, instead of DgaR, to complete the PTS cascade; but in the absence of D-glucosaminate, DgaR is phosphorylated by EII (DgaR-P2), which inhibits DgaR activation (Figure 1C; Miller et al., 2013).

*S*. Typhimurium can utilize D-glucosaminate as both a carbon and nitrogen source (Miller et al., 2013), so it is likely that this PTS system gives *S*. Typhmurium a competitive advantage over competing microbes under nutrient-limited conditions; the source of D-glucosaminate in the environment/host is likely to be other bacteria containing D-glucosaminate in lipid A or glucose oxidase that effectively oxidizes D-glucosamine (Miller et al., 2013).

#### GfrR

GfrR activates σ^54^-dependent transcription of the *gfrABCDEF* operon (Miller et al., 2015). GfrR differs from DgaR and other LevR-like bEBPs in its regulatory domain by substitution with tyrosine of the conserved histidine that is normally phosphorylated by HPr~P. By analogy to another LevR-like bEBP, MtlR (Joyet et al., 2015), GfrR is likely controlled solely by the repressive EII-mediated phosphorylation of PRD2; this results in GfrR being insensitive to the catabolite repression observed for DgaR (Miller et al., 2013), in which EI and HPr phosphorylation activity is directed to the uptake of another primary carbon source (glucose) instead of phosphorylation of the bEBP. Thus, *S.* Typhimurium is able to utilize glucose and fructoselysine (or glucoselysine) simultaneously (Miller et al., 2015).

Enzymes encoded by the *gfrABCDEF* operon enable glucoselysine and fructoselysine uptake and catabolism. Glucoselysine and frustoselysine, as well as other Maillard reaction products, are found at varying levels in the gut of human and animal hosts depending on the diet and microbiota (reviewed in Tuohy et al., 2006). The PTS permease and dual deglycases encoded by *gfrABCDEF* give *S*. Typhimurium flexibility in carbon and nitrogen sources, improving persistence in animal hosts (Chaudhuri et al., 2013).

### Signal sensing through ligand binding

In *S.* Typhimurium there are four bEBPs that are known, or predicted, to be regulated by the binding of an effector molecule to the regulatory domain: NorR, FhlA, PrpR, and RtcR. Although the regulatory domain structure is different for each of these bEBPs, in each case ligand binding alters the bEBP structure such that repression of AAA+ domain oligomerization, ATPase activity, and/or interaction with σ^54^ by the regulatory domain is relieved (Figure 1D).

#### NorR

NorR stimulates expression of nitric oxide (NO) reductase genes, *norVW*, in response to NO under anaerobic conditions (Gardner et al., 2003) The N-terminal region of NorR contains a GAF (cyclic GMP-specific and stimulated phosphodiesterases, *Anabaena* adenylate cyclases, and *E. coli* FhlA) domain with a non-heme iron center that recognizes NO (D'Autréaux et al., 2005). Binding of NO to the GAF domain relieves repression of the ATPase activity of the AAA+ domain, allowing activation of transcription from the σ^54^-dependent promoter for *norVW* (D'Autréaux et al., 2005). NorR recognizes three enhancer sequences upstream of the *norVW* operon, all of which are required for transcriptional activation (Tucker et al., 2010). As illustrated in Figure 1D, unlike many bEBPs, NorR is able to multimerize in the absence of the activating ligand, forming hexamers through assembly of dimers that are bound to the enhancer sequences. The hexamer-enhancer complex is unable to hydrolyze ATP until activated by NO binding (Bush et al., 2015). It has been suggested that this “pre-activated” complex may exist to enable rapid response to the presence of NO (Bush et al., 2015). NO and other reactive nitrogen species are generated by macrophages during the immune response to infection and have bactericidal and bacteriostatic effects on *Salmonella* (Vazquez-Torres et al., 2000). Transient increased sensitivity of a *norV* mutant to NO suggests that the NorR-regulated NO reductase is part of a multiple enzyme response to NO stress during the infection process (Mills et al., 2005).

#### RtcR

RtcR controls σ^54^-dependent transcription of putative RNA repair operons of *S*. Typhimurium (*rsr-yrlBA-rtcBA;* Chen et al., 2013; Samuels, 2014) and *E. coli* (*rtcBA*; Genschik et al., 1998; Engl et al., 2016). *rtcB* and *rtcA* encode homologs of the metazoan and archaeal RNA ligase and RNA 3′-phosphate cyclase, respectively (Das and Shuman, 2013). *rsr* and *yrlBA* of *Salmonella* encode homologs of metazoan Ro60 and Y-RNAs that form ribonucleoprotein complexes involved in noncoding-RNA quality control (Chen et al., 2013; Wolin et al., 2013). The regulatory domain of RtcR exhibits significant sequence similarity with the CRISPR-associated Rossmann fold (CARF) domains (Makarova et al., 2014). CARF domains are predicted to bind nucleotides, but the RtcR regulatory domain lacks a positively-charged residue involved in nucleotide binding (Makarova et al., 2014). The lack of this residue suggests that RtcR utilizes a different ligand, possibly a nucleoside or modified nucleotide (Makarova et al., 2014).

Metazoan RtcB functions in repair of *xbp*-1 mRNA, which is required for the unfolded protein response (Jurkin et al., 2014), as well as tRNA splicing (Popow et al., 2011). RtcA repairs 3′-phosphate or 2′-phosphate ends of cleaved RNA to 2′,3′-cyclic phosphates, which can serve as substrates for RtcB-mediated ligation (Remus and Shuman, 2013). RtcB and RtcA from *E. coli* exhibit the same biochemical activities as the metazoan homologs *in vitro* (Genschik et al., 1998; Tanaka et al., 2011). In addition, *E. coli* RtcB and RtcA utilize DNA substrates; RtcB adds a guanylyl “cap” to a 3′-phosphate end of nicked DNA (Das et al., 2013, 2014), and RtcA adenylylates DNA 5′-phosphate ends (Chakravarty and Shuman, 2011). In *S.* Typhimurium, the Rsr-YrlA complex associates with PNPase (polynucleotide phosphorylase; Chen et al., 2013); this is consistent with the activity of Rsr in *Deinococcus radiodurans*, where Rsr forms a ribonucleoprotein complex with YrlA and PNPase and is involved in starvation-induced rRNA degradation (Wurtmann and Wolin, 2010). Additionally, Rsr works with RNase PH and RNase II to fully process 23S rRNAs during growth at elevated temperature (37°C; Chen et al., 2007).

RtcR is activated in *S.* Typhimurium upon exposure to the antibiotic mitomycin C (MMC), stimulating transcription of the *rsr-yrlBA-rtcBA* operon (Samuels, 2014). MMC is an alkylating agent that causes intra- and inter-strand crosslinking in nucleic acids, and results in the formation of DNA-MMC (Bizanek et al., 1992) and RNA-MMC adducts (Snodgrass et al., 2010). MMC induces the SOS response (Kenyon and Walker, 1980), and RtcR activation by MMC is RecA-dependent, suggesting involvement of the SOS response in the activation of RtcR (Samuels, 2014). In *E. coli* RtcR is activated by conditions that disrupt translation, including VapC-mediated cleavage of tRNA^fmet^ and treatment with tetracycline (Engl et al., 2016). The signal that is recognized by RtcR in either bacterium is unknown, but candidate signal molecules include: alkylated bases or DNA-MMC adducts removed by nucleotide excision repair in the SOS response (reviewed in Kisker et al., 2013); MMC-modified nucleotides from rRNA or increased free nucleotide/nucleoside intracellular pools upon MMC-induced rRNA degradation (Suzuki and Kilgore, 1967a,b); 2′,3′-cyclic NMPs released from RNAs cleaved by toxins of toxin-antitoxin systems, which leave 2′,3′-cyclic phosphate at the 3′-end of cleaved RNA (reviewed in Sofos et al., 2015); or modified nucleotides of tRNAs (reviewed in Motorin and Helm, 2010) released by cleavage/degradation. The substrates for RtcA, RtcB, and Rsr-YrlA/B are unidentified in both *S*. Typhimurim and *E. coli*, although ribosome analysis in an *E. coli rtcB* mutant suggests a role in 16s rRNA stability (Engl et al., 2016).

## Conclusion

The σ^54^ regulon of *S.* Typhimurium is involved in a range of potential stress responses, including nitrogen/carbon limitation, cell envelope stress, nitric oxide stress, and nucleic acid damage/turnover. As summarized in this mini-review, the response to these stresses and the resulting modulation of the *S*. Typhimurium lifestyle are often mediated through bEBPs, which receive signals from the environment through a variety of mechanisms and activate the appropriate components of the σ^54^ regulon. Further characterization of RtcR activation by nucleic acid damage/modification and of the three currently uncharacterized bEBPs (STM0571, STM0652, and STM2361) will give a clearer picture of how bEBPs can alter the lifestyle of *S*. Typhimurium and other pathogens to improve their chances of survival during the infection process.

## Author contributions

CH, AK, and DS each made substantial intellectual contributions to the work, participated in the writing of the mini-review, and approved it for publication.

## Funding

NSF (MCB-1051175) and NIH (R21 AI117102-01A1) grants (to ACK) funded the study of the *Salmonella* RpoN regulon and putative RNA repair operon, respectively.

### Conflict of interest statement

The authors declare that the research was conducted in the absence of any commercial or financial relationships that could be construed as a potential conflict of interest.
